# Species Interactions Shape Nitrogen Utilization Characteristics and Influence Soil Quality in Jujube–Alfalfa Intercropping System

**DOI:** 10.3390/plants14132048

**Published:** 2025-07-03

**Authors:** Hang Qiao, Hui Cheng, Tiantian Li, Wenxia Fan, Yaru Zhao, Zhengjun Cui, Jinbin Wang, Qingqing Yang, Chengze Jia, Wei Zhang, Guodong Chen, Sumei Wan

**Affiliations:** 1 College of Agriculture, Tarim University, Alar 843300, China; 2 Key Laboratory of Genetic Improvement and Efficient Production for Specialty Crops in Arid Southern Xinjiang of Xinjiang Corps, Tarim University, Alar 843300, China; 3 Science and Technology Development Promotion Center, Xinjiang Production and Construction Crops, Urumqi 830002, China; 4 College of Agriculture, Shihezi University, Shihezi 832003, China

**Keywords:** legume intercropping, soil quality, nitrogen utilization efficiency, competitive ratio

## Abstract

Intercropping legumes offers a sustainable approach to enhance resource efficiency and yields, yet the effects of different legume densities and nitrogen addition levels on soil quality within such systems remain unclear. We conducted a comparative analysis of crop yield, nitrogen use efficiency, and soil quality between intercropping and monoculture systems, and further examined the effects of four planting densities (D1: 210 kg ha^−1^, six rows; D2: 280 kg ha^−1^, eight rows; D3: 350 kg ha^−1^, ten rows) and four nitrogen application levels (N0: 0 kg ha^−1^; N1: 80 kg ha^−1^; N2: 160 kg ha^−1^; N3: 240 kg ha^−1^) within a jujube–alfalfa (*Ziziphus jujuba* Mill. and *Medicago sativa* L. respectively) intercropping system. The results showed that intercropping significantly enhanced land productivity within the agricultural system, with the highest yields (alfalfa: 13790 kg ha^−1^; jujube: 3825 kg ha^−1^) achieved at an alfalfa planting density of 280 kg ha^−1^. While the intercropping systems generally improved productivity, an alfalfa planting density of 350 kg ha^−1^ resulted in an actual yield loss due to excessive nutrient competition at higher densities. As the planting density of alfalfa increased, its competitive ratio declined, whereas the competitive ratio of jujube trees increased. Compared to monocropping systems, intercropping systems demonstrated a clear trend of enhanced nitrogen utilization efficiency and improved soil quality, particularly at an alfalfa planting density of 280 kg ha^−1^. At an alfalfa density of 280 kg ha^−1^, the intercropping system exhibited increases of 15.13% in nitrogen use efficiency (NUE), 46.60% in nitrogen partial factor productivity (NPFP), and 32.74% in nitrogen nutrition index (NNI), as well as improvements in soil quality of 19.53% at a depth of 0–20 cm and 15.59% at a depth of 20–40 cm, compared to the monoculture system. Further analysis revealed that nitrogen utilization efficiency initially increased and then decreased with a rising competitive ratio of alfalfa. Accordingly, soil quality was improved along with the enhanced nitrogen utilization efficiency. Thus, at an alfalfa planting density of 280 kg ha^−1^, resource use efficiency and soil quality were maximized as a result of optimal interspecific competitiveness and the highest nitrogen use efficiency, with minimal influence from the application of nitrogen fertilizer.

## 1. Introduction

Soil quality is a fundamental factor in ensuring food security, safeguarding human health, and promoting the sustainable development of ecological systems [1]. Soil quality assessment involves the systematic monitoring and evaluation of soil properties, functions, and conditions vital for maintaining ecosystem performance [2]. Higher soil quality fosters stable biological communities, optimal soil fertility, well-structured soil, and favorable moisture conditions [3]. Therefore, the evaluation of soil quality is paramount for achieving sustainable soil management and improving agricultural productivity.

Nitrogen (N) is a vital nutrient for crop growth, and its use efficiency reveals crucial insights into N demand and supply. The critical nitrogen (Nc) dilution curve is frequently employed to evaluate the allometric growth relationship between N uptake dynamics and biomass accumulation [4]. Additionally, the Nc dilution curve underpins the calculation of the N nutrition index (NNI), which quantifies crop yield, quality, and nitrogen demand, with NNI > 1 indicating a N surplus, NNI = 1 representing N sufficiency, and NNI < 1 signifying a N deficit [5]. Thus, assessing the Nc dilution curve and NNI in real-time can effectively monitor crop N demand and supply.

Interspecific competition serves as one of the key yield advantages in intercropping systems [6]. For example, the yield advantage in wheat/vetch intercropping was largely attributed to the superior competitive ability of wheat [7]. For leguminosae/gramineae intercropping systems, legume crops generally exhibited a lower competitive advantage due to the stronger competitiveness of cereal crops [8]. In some specific intercropping systems, legumes demonstrated a strong competitive ability, which can be linked to enhanced nutrient acquisition facilitated by shading effects from the accompanying intercrop species [9]. Despite these discrepant patterns of interspecific competition, improved yield was observed. However, declining productivity for soybean was observed in the maize/soybean system. Thus, understanding crop competitive advantages in specific intercropping systems is essential for accurately assessing their productivity.

The jujube tree (*Ziziphus jujuba* Mill.) is a key economic crop in Xinjiang, with a cultivation area of about 3.2 × 10^4^ ha, playing a pivotal role in regional development. In addition to its economic value, it provides important ecological benefits in southern Xinjiang, such as windbreaks, sand fixation, and ecological improvement. However, prolonged monoculturing has resulted in soil degradation and increased pest and disease incidence, adversely affecting both the health and productivity of jujube trees. Furthermore, the lack of scientific management—particularly in fertilization and nutrient balance—has led to low yields and poor fruit quality, reducing overall production efficiency. Therefore, optimizing planting structures and improving fertilization practices are essential to enhance soil quality and resource utilization, thereby promoting the sustainable development of the jujube industry in Xinjiang. Intercropping legumes with other crops (e.g., maize, apple) offers a transformative approach to shift from the current resource-intensive and inefficient monocropping systems to sustainable, resource-efficient, legume-based intercropping systems [10]. Such systems enable both crops to more effectively capture and utilize available resources, resulting in higher yields while minimizing environmental impacts [11]. However, in legume-based intercropping systems, the effects of varying legume planting densities and fertilization rates on system productivity remain insufficiently studied. Alfalfa (*Medicago sativa* L.) is one of the most important leguminous forage crops in China, and it plays a crucial role in improving soil structure, enhancing soil fertility, and reducing greenhouse gas emissions [12].

This study aims to systematically examine the effects of alfalfa planting density and varying nitrogen application rates on soil quality and nutrient use efficiency in jujube–alfalfa intercropping systems. Specifically, the objectives are: (1) to evaluate the impact of different alfalfa planting densities on soil quality and (2) to assess how varying nitrogen fertilization levels regulate nitrogen cycling efficiency within the system. We hypothesized that (i) appropriate N application in legume–jujube intercropping systems could improve soil quality and N use efficiency by mitigating competition between crops and microorganisms at low N levels and avoiding diminishing marginal returns at excessive nitrogen concentrations [13]; and (ii) legume–jujube intercropping systems with a lower planting density of alfalfa would have higher yield, soil quality and N use efficiency due to the increased competitive effects with increased planting density [14].

## 2. Results

### 2.1. Yield and Competitive Ratio

**Figure 1:**
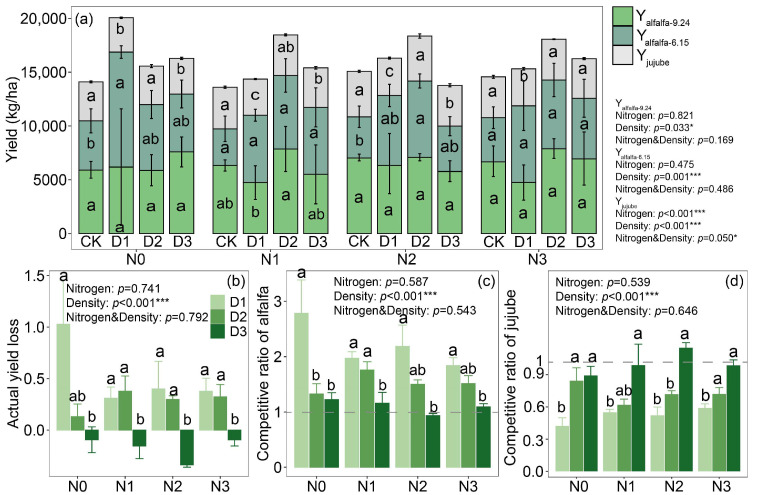
Yield characteristics of the jujube–alfalfa intercropping system: (**a**) total yield, (**b**) actual yield loss; crop competitive ratio within the intercropping system: (**c**) alfalfa (*Medicago sativa* L.), (**d**) jujube (*Ziziphus jujuba* Mill.). Notes: D1, D2, and D3 represent jujube–alfalfa intercropping systems with planting density of alfalfa at 210, 280 and 350 kg ha^−1^, respectively. CK represents monoculture systems of alfalfa and jujube; N0, N1, N2, and N3 represent different fertilization levels of 0, 80, 160 and 240 kg ha^−1^, respectively. In panel a, Y_alfalfa-6.15_ refers to the yield of the first alfalfa harvest, which is represented by dark green in the figure, while Y_alfalfa-9.24_ corresponds to the yield of the second harvest within the same year, shown in light green. Y _jujube_ represents the annual yield of jujube, indicated by the gray section in the figure. Different lowercase letters indicate significant differences in density (*p* < 0.05). *, **, and *** indicate significant effects of nitrogen application, planting density, and their interaction at 0.01 < *p* ≤ 0.05, 0.001 < *p* ≤ 0.01, and *p* ≤ 0.001, respectively.

Compared with the monoculture system, jujube tree yield in the intercropping system tended to decrease by 7%, whereas alfalfa yield tended to increase by 19% under intercropping (Table S3). In the intercropping systems, only planting density significantly affected alfalfa yield, whereas planting density, nitrogen application rate, and their interaction all had significant effects on jujube tree yield (*p* < 0.05) (Figure 1a). At an alfalfa planting density of 280 kg ha^−1^, the yield reached its maximum, averaging 13,789.50 kg ha^−1^. For jujube trees, the highest yield was observed at the same planting density, with an average of 3825 kg ha^−1^, and at a nitrogen application rate of 160 kg ha^−1^, with an average yield of 3912.5 kg ha^−1^.

Significant intercropping advantages were observed in the alfalfa–jujube system at planting densities of 210 kg ha^−1^ and 280 kg ha^−1^, while these advantages disappeared when the density increased to 350 kg ha^−1^ (Figure 1b). In the jujube–alfalfa intercropping system, alfalfa consistently exhibited a competitive ratio greater than 1, while that of jujube remained below 1, highlighting the significantly stronger competitive ability of alfalfa compared to jujube. Moreover, as alfalfa planting density increased, its competitiveness declined, whereas the competitive ability of jujube correspondingly improved (Figure 1c,d).

### 2.2. Nitrogen Uptake, N Use Efficiency and Soil Quality

**Figure 2:**
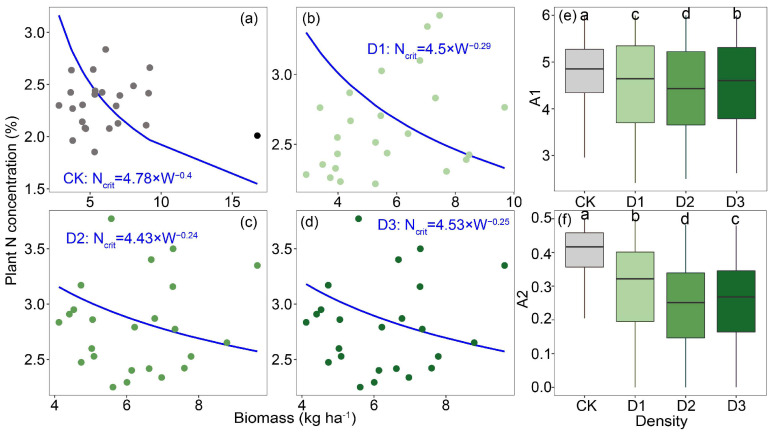
Fitted critical nitrogen concentration dilution curves for alfalfa based on Bayesian statistical methods in (**a**) monoculture system, (**b**) intercropping system with planting density at 210 kg ha^−1^, (**c**) intercropping system with planting density at 280 kg ha^−1^, (**d**) and intercropping system with planting density at 350 kg ha^−1^; differences in parameters (**e**) A1 and (**f**) A2. Notes: Different lowercase letters indicate significant differences in density (*p* < 0.05).

Nc dilution curves for alfalfa were generated using a Bayesian statistical model across varying planting densities. The Nc dilution curve derived from the Bayesian model revealed that the values in the intercropping system were significantly higher than those in the monoculture alfalfa system, displaying a trend of first decreasing and then increasing with rising planting density (Figure 2a–d). Additionally, parameters A1 and A2 in the monoculture alfalfa system, as estimated by the Bayesian model, were also lower than those in the intercropping system (Figure 2e,f).

**Figure 3:**
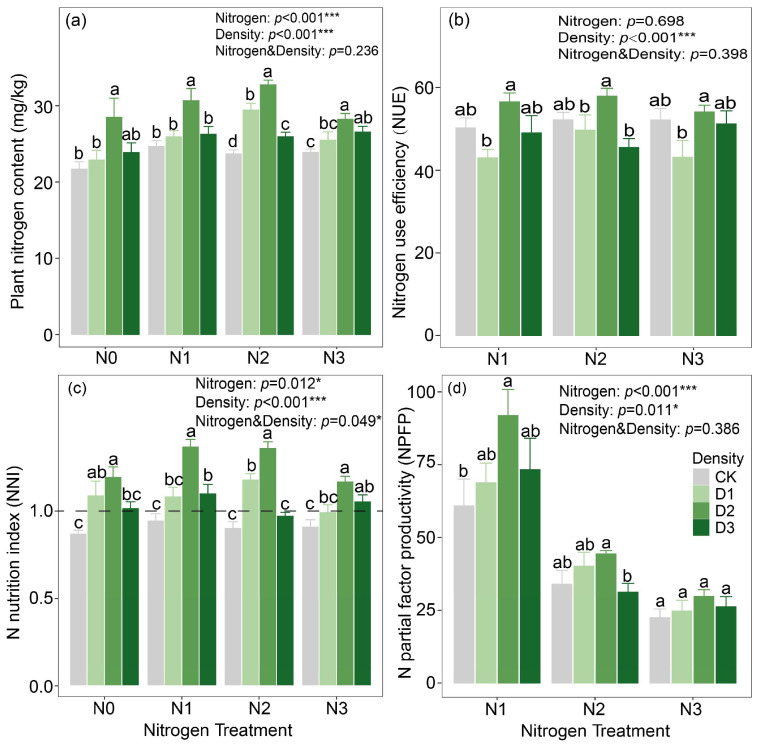
(**a**) Plant nitrogen content, (**b**) nitrogen use efficiency, (**c**) nitrogen nutrition index and (**d**) nitrogen partial factor productivity of alfalfa. Notes: Different lowercase letters indicate significant differences in density (*p* < 0.05). *, **, and *** indicate significant effects of nitrogen application, planting density, and their interaction at 0.01 < *p* ≤ 0.05, 0.001 < *p* ≤ 0.01, and *p* ≤ 0.001, respectively.

Compared with the monoculture system, the intercropping system increased plant nitrogen content by 13.68%, NNI by 25.24%, and NPFP by 42.02%. Although the intercropping system also enhanced NUE relative to the monoculture system, the difference between the two systems was not statistically significant (Table S4). Both planting density and nitrogen application significantly affected plant nitrogen content, NPFP, and NNI, with their interaction also exerting a significant impact on NNI (*p* < 0.05). By contrast, only planting density had a significant effect on NUE (Figure 3). At a planting density of 280 kg ha^−1^, plant nitrogen content, NUE, and NNI reached their highest values, with mean values of 30.02 mg kg^−1^, 57.65%, and 1.14, respectively. Likewise, NPFP also attained its maximum at this planting density.

**Figure 4:**
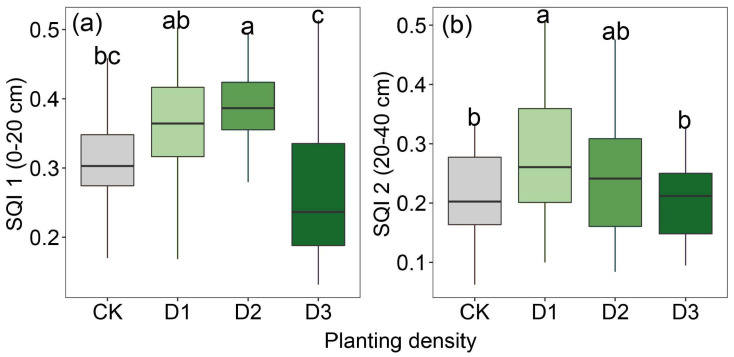
Soil quality in alfalfa monoculture system and jujube–alfalfa intercropping system at (**a**) 0–20 cm and (**b**) 20–40 cm. Notes: Different lowercase letters indicate significant differences in density (*p* < 0.05).

Compared to the monoculture system, the intercropping system showed a pronounced improvement in soil quality. In the 0–20 cm soil layer, the highest soil quality was recorded at a planting density of 280 kg ha^−1^, which was 8.71% higher than at 210 kg ha^−1^, 30.96% higher than at 350 kg ha^−1^, and 19.53% higher than in the monoculture system. In the 20–40 cm soil layer, there was no significant difference in soil quality between the 210 kg ha^−1^ and 280 kg ha^−1^ planting densities; however, soil quality at 210 kg ha^−1^ was 25.30% higher than in the monoculture system, while that at 280 kg ha^−1^ was 15.59% higher (Figure 4).

### 2.3. Relationships Between Soil Environmental Factors, Functional Microorganisms, and Soil Quality and Competitive Ratio

**Figure 5:**
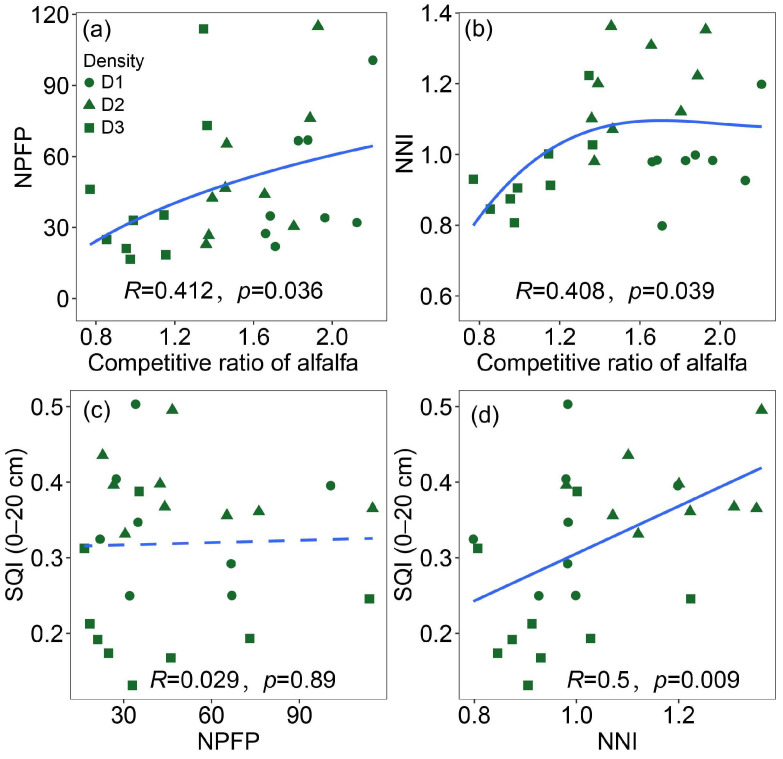
Regression analyses of the relationships between the competitive ratio of alfalfa and NPFP (**a**) and NNI (**b**), as well as between NPFP and SQI (**c**), and NNI and SQI (**d**). Notes: Solid lines indicate significant correlations, while dashed lines indicate non-significant correlations (*p* < 0.05).

Regression analysis revealed that the competition ratio of alfalfa is logarithmically correlated with NPFP and exhibits a binary regression relationship with NNI. Both NPFP and NNI demonstrated a clear trend of initially increasing and subsequently decreasing as the alfalfa competitive ratio rose (Figure 5a,b). A comprehensive analysis of the relationships between NPFP, NNI, and soil quality revealed that, while NPFP had no significant impact on soil quality, NNI exhibited a robust and significantly positive correlation with soil quality (Figure 5c,d).

## 3. Discussion

### 3.1. Effects of Planting Density on Yield and Competitive Ratio in Intercropping Systems

Intercropping systems that integrate jujube trees with alfalfa offer a notable advantage over monoculture systems by significantly enhancing overall land productivity. The yield advantage of the intercropping system is attributed to the efficient resource utilization enabled by the complementary spatial arrangement of the aboveground parts of jujube and alfalfa. However, the impact of intercropping on individual jujube tree and alfalfa crop yields can vary, with intercropping not always leading to yield improvements and, in some cases, resulting in yield reductions. While jujube–alfalfa intercropping systems effectively optimize the utilization of light and thermal resources, they may also intensify competition for soil nutrients, posing challenges for resource allocation [15]. Notably, the highest yield under intercropping was achieved at a planting density of 280 kg ha^−1^, which disagreed with hypothesis ii. This trend can be explained by alfalfa’s nitrogen-fixing ability, which promotes nutrient utilization by companion crops [16], thereby enhancing yield at a planting density of 210 kg ha^−1^ to 280 kg ha^−1^. However, with a further increase in planting density to 350 kg ha^−1^, intensified intraspecific competition within the alfalfa population could allocate the nitrogen-fixed nutrients toward its own growth, while simultaneously competing with jujube trees for vital resources. Additionally, excessive alfalfa density could lead to reduced photosynthetic efficiency and consequently lower yield. Confusingly, variations in N application rates did not appear to affect the aforementioned indicators, which is entirely inconsistent with hypothesis i, highlighting the need for further investigation to uncover the underlying reasons behind this outcome.

To better understand the competitive dynamics between alfalfa and jujube trees within the intercropping system, we found that the competitive ratio of alfalfa was typically greater than 1, whereas that of jujube trees consistently remained below 1. This disparity may be explained by alfalfa’s traits as a leguminous plant, which allow it to achieve higher rates of absorption or transformation of essential nutrients, such as N and P, thereby granting it a competitive edge in acquiring soil resources [17]. Interestingly, we found that as the planting density of alfalfa increased, its competitive ratio declined, while that of jujube increased. This phenomenon may be attributed to the increased canopy density of alfalfa at higher planting densities, which limits light penetration to the leaves and reduces photosynthetic efficiency, coupled with root overlap in constrained spaces that likely hampers nutrient absorption efficiency [18]. These combined factors intensify intraspecific competition within alfalfa, resulting in a density dependence effect. According to the Mitscherlich model, the nonlinear relationship between density and resource competition suggests that when alfalfa density exceeds a certain threshold, its per-plant resource acquisition declines significantly [19]. Conversely, the competitiveness of jujube trees increased, which might be attributed to the variable responses of different crops to planting density. Some crops, such as alfalfa, may experience adverse effects under high-density conditions, whereas others may respond positively. This phenomenon was demonstrated in the study by Manntschke et al. [20], which found that the competitiveness of wheat could be significantly enhanced at higher planting densities.

### 3.2. N Utilization Characteristics of Alfalfa and Soil Quality in Intercropping Systems

Nc dilution curves for alfalfa under varying planting densities were established using a Bayesian statistical approach, finding that, within the intercropping system, alfalfa at densities of 210 kg ha^−1^ and 350 kg ha^−1^ exhibited significantly higher Nc values than at a density of 280 kg ha^−1^. This phenomenon might partially reflect differences in alfalfa’s capacity to acquire and utilize N, which are influenced by planting density and its cascading effects on resource competition, root architecture, and overall plant physiological processes [21]. Furthermore, the differences in Nc dilution curve parameters A1 and A2 under different planting densities were analyzed. Analysis of Nc dilution curve parameters A1 and A2 under different planting densities revealed that both parameters reached their lowest values at a planting density of 280 kg ha^−1^. Specifically, A1 represents the critical N concentration when the aboveground biomass is 1 t ha^−1^, while A2 indicates the rate at which N concentration decreases with increasing biomass [22]. At low planting density, abundant N availability negates the need for alfalfa to activate a high N-use efficiency strategy; however, as planting density increases, alfalfa adopts a high-efficiency N utilization strategy by modifying the rhizosphere microenvironment to enhance N use efficiency [23]. For example, alfalfa modifies the composition of root exudates, such as organic acids and enzymatic activities, to mobilize insoluble N in the soil while simultaneously enhancing N allocation efficiency to grains and stems by stimulating photosynthetic C metabolism, such as through increased ribulose-1,5-bishosphate carboxylase enzyme activity [24,25]. This explanation also illustrates the high NNI, NUE and NPFP observed in alfalfa under the 280 kg ha^−1^ planting density. To quantify soil quality under different planting densities of alfalfa, we found that the planting density of 280 kg ha^−1^ resulted in significantly higher soil quality compared to other treatments, consistent with the previously observed N utilization characteristics of alfalfa. At this optimal planting density, the distribution of alfalfa root density improved soil physical structure, increased root biomass, enhanced soil porosity and water-holding capacity, maintained soil aeration and looseness, and promoted rhizosphere biomass accumulation through root-secreted carbohydrates.

### 3.3. Optimal Competitiveness of Alfalfa Facilitates Soil Quality by Modifying N Utilization Indices

In our analysis, we found that an appropriate competition ratio for alfalfa can enhance its N utilization indices, thereby further improving soil quality. For example, some plants can increase their uptake of ammonium ions (NH_4_^+^) during competition to maintain their competitive advantage [26]. Additionally, leguminous species enhance nitrogen use efficiency by forming symbiotic relationships with rhizobia, thereby fixing atmospheric nitrogen and converting it into forms readily available to plants [27]. Additionally, interspecific competition can promote dynamic changes in soil nitrogen by stimulating soil microbial activity and accelerating the mineralization of organic matter, thereby increasing nitrogen availability and ultimately enhancing soil nutrient content and quality [23]. Conversely, several studies have reported that root competition among plants can significantly reduce the efficiency of soil nitrogen uptake—for example, nitrogen uptake may decrease to as low as 57% under root competition, indicating that root competition has a much stronger inhibitory effect on nitrogen uptake, which may subsequently promote nutrient accumulation in the soil and ultimately enhance soil quality [15].

Appropriate levels of competition also exemplify the complementary and facilitative ecological principles underlying intercropping systems [8]. Rational spatial niche differentiation is achieved, as alfalfa and jujube trees utilize light and heat resources at varying heights—jujube trees reach 1.5–2 m, while alfalfa remains below 0.5 m [28]. Additionally, the differentiation in root distribution enhances this complementary resource utilization mechanism: jujube tree roots are primarily distributed at depths of 0–40 cm, whereas alfalfa roots are found at 0–20 cm [29]. This synergistic resource utilization strategy confers a sustainable advantage, promoting the long-term development of the jujube–alfalfa intercropping system. Leguminous plants enhance nutrient utilization through biological N fixation and the release of protons and enzymes in the rhizosphere, which promote the mineralization of organic P, thereby directly or indirectly increasing soil nutrient availability and strengthening the facilitative mechanisms of intercropping systems. These complementary and facilitative effects enhance the diversity of organic matter sources and the storage of available nutrients in the soil, thereby improving overall soil quality.

## 4. Materials and Methods

### 4.1. Site Description and Experimental Design

The study was conducted at an experimental field located at Tarim University in Alar, Xinjiang (40°32′34″ N, 81°18′07″ E). The study site is characterized by a warm-temperate, extremely continental, arid desert climate, with an average annual sunshine duration of approximately 2800 h and a sunshine percentage of 60%. Precipitation is scarce—mean annual precipitation is 60 mm, with little snowfall in winter—while surface evaporation is intense, reaching 2200 mm annually. The soil at the site has a sandy-loamy texture and is classified as Aridisols. The initial soil properties (0–20 cm) at the start of the experiment were as follows: pH 7.86, organic carbon (SOC) of 5.47 g kg^−1^, alkali-hydrolyzed nitrogen (AN) of 63 mg kg^−1^, available phosphorus (AP) of 32.55 mg kg^−1^, and available potassium (AK) of 107.33 mg kg^−1^. For the subsoil (20–40 cm), the properties were as follows: pH 7.91, SOC content of 5.17 g kg^−1^, AN of 47.83 mg kg^−1^, AP of 29.93 mg kg^−1^, and AK of 103.26 mg kg^−1^.

The experiment (2021–2022) evaluated three cropping systems: alfalfa (*Medicago sativa* L.) monoculture, jujube (*Ziziphus jujuba* Mill.) monoculture, and jujube–alfalfa intercropping. The alfalfa variety employed was Zhongmu No. 1, while the jujube variety utilized was Junzao. In the intercropping system, a two-factor split-plot design was used. The main plot included four nitrogen levels: N0, N1, N2, and N3 (0, 80, 160, and 240 kg ha^−1^), while the subplot included three alfalfa planting densities: D1 (210 kg ha^−1^ sowing rate; 6 rows), D2 (280 kg ha^−1^ sowing rate; 8 rows), and D3 (350 kg ha^−1^ sowing rate; 10 rows) based on previous studies. Jujube trees were spaced 3 m × 1 m, with alfalfa intercropped between rows, and the nearest alfalfa row 0.5 m from the trees. In monoculture cropping systems, only nitrogen levels were varied. In both monoculture and intercropping systems, each plot contained 30 jujube trees. Each treatment was replicated three times in a randomized design. Plot size was 42 m^2^ (length 14 m × width 3 m). The jujube in both the jujube–alfalfa intercropping system and the jujube monoculture system was planted in 2012, while the alfalfa in the jujube–alfalfa intercropping and alfalfa monoculture systems was planted in 2021. The jujube trees were harvested annually in October, while alfalfa aboveground biomass was collected in June and September each year. The nitrogen fertilizer applied was urea (N content: 46%), which was divided into four applications corresponding to the different growth stages of jujube trees, at a ratio of 1:3:3:2. Drip irrigation was employed, and field management practices—including weeding and pest control—were conducted in accordance with standard field protocols and applied uniformly across all treatments. In the subsequent data analysis, the alfalfa monoculture system was used as the control (CK) to evaluate and compare the yield and nitrogen utilization characteristics of alfalfa within the intercropping system.

### 4.2. Determination of Yield, Actual Yield Loss and Competitive Ratio

Alfalfa was harvested on 15 July and 24 September to evaluate its yield, with a 1 m × 1 m quadrant sampled for each treatment, leaving a stubble height of 5 cm. The samples were dried at 105 °C for 0.5 h, followed by oven drying at 70 °C to a constant weight, and the yield was calculated and expressed on a per-hectare basis. At the full-ripening stage of jujube, five trees were randomly selected from each treatment to measure the yield per tree. The average yield was then calculated and converted to a per-hectare basis.

Actual yield loss and competitive ratio within the intercropping system were calculated as follows [30,31]:

(1) $$Y=\left(\frac{Y_{\mathrm{ai}}/Z_{\mathrm{ai}}}{Y_{a}/Z_{a}}\right)+\left(\frac{Y_{\mathrm{ji}}/Z_{\mathrm{ji}}}{Y_{j}/Z_{j}}\right)-2$$

(2) $$C_{a}=\left(\frac{Y_{\mathrm{ai}}Y_{j}}{Y_{a}Y_{\mathrm{ji}}}\right)\times \left(\frac{Z_{\mathrm{ji}}}{Z_{\mathrm{ai}}}\right)$$

(3) $$C_{j}=\left(\frac{Y_{\mathrm{ji}}Y_{a}}{Y_{j}Y_{\mathrm{ai}}}\right)\times \left(\frac{Z_{\mathrm{ai}}}{Z_{\mathrm{ji}}}\right)$$

where Z_a_ and Z_j_ are the planting proportions of alfalfa and jujube trees in the monocropping system, respectively, and Z_ai_ and Z_ji_ are their proportions in the intercropping system. Similarly, Ya and Yj are the yields of alfalfa and jujube in monocropping, while Y_ai_ and Y_ji_ are their yields in intercropping. Y represents the actual yield loss in the alfalfa–jujube intercropping system, where Y > 0 indicates an intercropping advantage, and Y < 0 signifies no yield advantage. C_a_ and C_j_ represent the competitive ratios of alfalfa and jujube trees in the intercropping system, respectively. C_a_ > 1 indicates that alfalfa is more competitive than jujube trees, while C_j_ > 1 indicates that jujube trees are more competitive than alfalfa.

### 4.3. Establishment of Critical Nitrogen Concentration Dilution Curves, Nitrogen Nutrition Index, Nitrogen Use Efficiency, and Partial Factor Productivity

A Bayesian hierarchical model was used to establish the Nc dilution curve, as outlined by Makowski et al. [4]. This model employs a linear-plus-plateau function to describe the relationship between nitrogen concentration and aboveground dry biomass. Unlike traditional methods, it eliminates the need to classify data into N-limited and non-limited groups, enabling unified estimation of the Nc dilution curve while accounting for the uncertainty of fitted curves. This approach also allows direct comparisons of Nc dilution curves under varying alfalfa densities. Weakly informative priors, following Makowski et al. [4], were defined as: μBMAX~N (6, 0.1), μS~N (0, 0.1), A1~Unif (2, 7), A2~Unif (0, 0.5), 1/σ^2^BMAX~Gamma (0.001, 0.001), 1/σ^2^S~Gamma (0.001, 0.001), 1/τ^2^b~Gamma (0.001, 0.001), 1/τ^2^n~Gamma (0.001, 0.001), and 1/τ^2^t~Gamma (0.001, 0.001). The MCMC algorithm was initially run with three chains of 50,000 iterations each, followed by an additional 100,000 iterations after confirming convergence to estimate coefficients A1 and A2 of the Nc dilution curve. This model was applied to varying alfalfa densities in jujube–alfalfa intercropping systems and monocropped alfalfa, yielding four Nc dilution curves. The variations in coefficients A1 and A2 across treatments were also systematically analyzed.

The nitrogen nutrition index (NNI) for alfalfa was determined by calculating the ratio of the actual nitrogen concentration (Na) to the critical nitrogen concentration (Nc) for each density, as follows:

(4) $$\mathrm{NNI}=\frac{\mathrm{Na}}{\mathrm{Nc}}$$

Nitrogen use efficiency (NUE, %) and nitrogen partial factor productivity (PFPN, kg·kg^−1^) for alfalfa were calculated using the following formulas [32]:

(5) $$\mathrm{NUE}=\frac{N\;\mathrm{uptake}\;\mathrm{in}\;\mathrm{fertilizer}\;\mathrm{treatment}\;-\;N\;\mathrm{uptake}\;\mathrm{in}\;\mathrm{the}\;\mathrm{control}}{N\;\mathrm{applicantion}\;\mathrm{amount}}\times 100\%$$

(6) $$\mathrm{NPFP}=\frac{\mathrm{yield}}{N\;\mathrm{applicantion}\;\mathrm{amount}}$$

### 4.4. Soil Sampling and Analysis

Soil samples were collected on 29 May, 15 June, 14 August, and 24 September from the inter-row areas of jujube-alfalfa intercropping, as well as from alfalfa monoculture systems, using the five-point sampling method. A soil auger was used to collect samples at two soil depths (0–20 cm and 20–40 cm), with three replicates per treatment. A portion of the soil samples was air-dried for analysis of soil nutrients, while another portion was sieved and stored at 4 °C for assessment of culturable soil microorganisms.

Soil pH was measured with a pH meter (Mettler Toledo FE28, Shanghai, China) at a soil-to-water ratio of 1:2.5. SOC was determined using the potassium dichromate oxidation method with external heating. AN was analyzed via the alkali-hydrolysis diffusion method. AP was quantified using sodium bicarbonate extraction and the molybdenum–antimony colorimetric method, while AK was measured via ammonium acetate extraction and flame atomic absorption spectrometry. The abundance of soil microorganisms was assessed using the plate counting method, with bacteria cultured on nutrient agar, fungi on Martin’s medium, and actinomycetes on Gause’s No. 1 medium.

### 4.5. Soil Quality Analysis

Soil quality index (SQI) was used to assess the impact of alfalfa planting density on soil quality by the method of Jahany & Rezapour [33]. SQI was established based on nine soil parameters, with each parameter scored (S_i_) on a scale from 0 to 1. Scores were determined using either Equation (7) (“more is better”, S_i_′) or Equation (8) (“less is better”, S_i_″), with pH evaluated using Equation (8) and all other indicators using Equation (7).

(7) $$S_{i}^{\prime }=\frac{x_{i}-x_{\min}}{x_{\max}-x_{\min}}$$

(8) $$S_{i}^{\prime \prime }=\frac{x_{\max}-x_{i}}{x_{\max}-x_{\min}}$$

where S_i_ (S_i_′ and S_i_″) represents the score of the i_th_ parameter, while x_i_, x_min_ and x_max_ denote the measured value, minimum value, and maximum value of the parameter i, respectively.

Second, the weight (W_i_) of the i_th_ parameter was determined through principal component analysis (PCA), calculated as the ratio of its commonality to the total commonalities of all parameters. SQI was calculated by the following equation:

(9) $$\mathrm{SQI}=\sum_{i=1}^{n}W_{i}\times S_{i}\;$$

where n is the number of soil parameters, and W_i_ and S_i_ are the weight and score of the i_th_ parameter.

### 4.6. Statistical Analysis

The effects of alfalfa planting density and N fertilizer treatments on yield, actual yield loss, competition ratio of alfalfa and jujube, N content of alfalfa, N use efficiency of alfalfa, N nutrition index (NNI), N partial factor productivity (NPFP), and A1/A2 indices of the N dilution curve were analyzed using one-way ANOVA. Two-way ANOVA was used to assess the combined effects of N application and planting density. Regression analysis explored the relationships between the alfalfa competition index and NPFP/NNI, as well as NPFP/NNI and soil quality. All statistical analyses were performed using R software (version 4.1.0).

## 5. Conclusions

In this study, the intercropping system demonstrated clear advantages over the monoculture systems in terms of yield, nitrogen use efficiency, and soil quality, highlighting that optimizing resource allocation and ecological interactions through intercropping can markedly enhance agricultural productivity. Specifically, when the alfalfa planting density was set at 280 kg ha^−1^ within the intercropping system, the yields of alfalfa and jujube reached 13,790 kg ha^−1^ and 3825 kg ha^−1^, respectively. At this density, plant nitrogen content (30.02 mg kg^−1^), nitrogen use efficiency (57.65%), nitrogen nutrition index (1.14), and soil quality (0.39) all reached their highest levels. Furthermore, moderate competition among crops in the intercropping system contributed to improved soil quality by optimizing nitrogen utilization. Overall, adopting an alfalfa planting density of 280 kg ha^−1^ in intercropping with jujube can be identified as a key strategy for promoting sustainable agricultural development and enhancing resource use efficiency in agroecosystems in southern Xinjiang.

## Data Availability

The original contributions presented in the study are included in the article/Supplementary Materials, further inquiries can be directed to the corresponding authors.

## Supplementary Materials

The following supporting information can be downloaded at: https://www.mdpi.com/article/10.3390/plants14132048/s1, Table S1: Variations of soil physicochemical properties and microbial quantity at different treatments; Table S2: Effect of nitrogen application, planting density and their interaction on soil physicochemical properties and microbial quantity; Table S3: Variations of yield in intercropping and monoculture systems; Table S4: Variations of plant nitrogen content, nitrogen use efficiency, nitrogen nutrition index and nitrogen partial productivity in intercropping and monoculture systems.
