# Supplementary material for: Species Interactions Shape Nitrogen Utilization Characteristics and Influence Soil Quality in Jujube–Alfalfa Intercropping System
Source: Plants (Basel). 2025 Jul 3;14(13):2048. doi: 10.3390/plants14132048 (PMC12251689; doi:10.3390/plants14132048)
Supplement: Supplementary file 1 [file plants-14-02048-s001.zip › plants-3581184-supplementary.pdf]

Table S1 Variations of soil physicochemical properties and microbial quantity at different treatments

Nitrogen application	Density	soil layer	pH	SOC (g/kg)	HN (mg/kg)	AK (mg/kg)	AP (mg/kg)	Actinomycetes	bacteria	fungi
N0	CK	0-20cm	7.91	2.76	45.5	149.08	36.47	7	3	5
		20-40cm	7.87	1.48	19.83	118.77	24.44	1	1	2
	D1	0-20cm	7.78	3.43	47.25	335.04	27.76	1	1	1
		20-40cm	7.81	2.88	30.92	295.92	29.84	0	1	0
	D2	0-20cm	7.85	3.65	47.25	244.13	39.32	2	1	2
		20-40cm	7.86	2.86	35	169.85	35.87	2	2	1
	D3	0-20cm	7.84	2.1	36.17	124.74	31.37	2	0	1
		20-40cm	7.87	1.1	18.67	109.87	36.03	0	0	1
N1	CK	0-20cm	7.87	4.47	47.84	153.38	47.8	2	2	3
		20-40cm	8.05	2.31	18.67	120.35	31.55	1	0	1
	D1	0-20cm	7.91	1.99	35.59	152.57	33.85	2	3	1
		20-40cm	7.83	1.37	56	140.49	35.5	1	0	1
	D2	0-20cm	7.73	3.05	45.5	133.44	37.93	5	1	2
		20-40cm	7.99	1.42	19.25	122.77	38.76	0	4	0
	D3	0-20cm	7.89	3.15	36.17	146.73	35.83	2	2	2
		20-40cm	7.84	1.96	29.17	136.71	38.56	2	1	2
N2	CK	0-20cm	7.85	4.3	40.84	182.78	31.69	1	1	5
		20-40cm	7.89	2.83	27.42	163.75	41.46	1	1	2
	D1	0-20cm	7.82	4.76	44.33	154.95	38.84	9	7	5
		20-40cm	8.02	3.54	31.5	131.37	33.21	1	0	2
	D2	0-20cm	7.77	4.92	42.58	168.64	39.84	1	1	7
		20-40cm	8.03	3.1	22.17	158.62	30.28	0	0	1
	D3	0-20cm	7.82	3.57	36.17	184.57	33.24	1	1	3
		20-40cm	7.91	2.51	19.84	219.9	32.21	0	0	1
N3	CK	0-20cm	7.88	3.57	37.92	187.3	37.19	2	2	4
		20-40cm	7.81	1.93	19.83	217.54	30.54	3	3	5
	D1	0-20cm	7.8	3.52	25.67	177.28	32.57	4	3	4
		20-40cm	7.91	2.18	23.92	168.78	31.26	1	1	2
	D2	0-20cm	7.74	4.59	49	234.69	29.98	4	2	6
		20-40cm	7.83	2.53	23.92	172.55	35.51	1	0	2
	D3	0-20cm	7.73	2.74	26.84	215.03	33.13	3	2	7
		20-40cm	7.84	1.83	31.5	201.39	32.33	1	0	2

Table S2 Effect of nitrogen application, planting density and their interaction on soil physicochemical properties and microbial quantity

		pH	SOC	HN	AK	AP	Actinomycetes	Bacteria	Fungi
0-20cm	Nitrogen	0.277	< 0.001***	0.133	< 0.001***	0.693	0.804	0.595	< 0.001***
	Density	0.029*	0.003**	0.015*	0.007**	0.708	0.115	0.034*	0.099
	Nitrogen&Density	0.398	0.017*	0.537	< 0.001***	0.931	< 0.001***	0.041*	0.014*
20-40cm	Nitrogen	0.008**	0.001**	0.630	< 0.001***	0.812	0.323	0.281	0.003**
	Density	0.498	0.121	0.041*	0.027*	0.899	0.546	0.099	0.081
	Nitrogen&Density	0.061	0.099	0.094	< 0.001***	0.934	0.070·	0.018*	0.746

Table S3 Variations of yield in intercropping and monoculture systems

	Intercropping system	Monoculture system	*p*
Yield of alfalfa (kg ha^{-1})	12,915.99	10,461.67	0.008
Yield of jujube (kg ha^{-1})	3602.78	3875.83	0.004

Table S4 Variations of plant nitrogen content, nitrogen use efficiency, nitrogen nutrition index and nitrogen partial productivity in intercropping and monoculture systems

	Intercropping system	Monoculture system	*p*
Plant nitrogen content (mg/kg)	27.19	23.47	< 0.001
Nitrogen use efficiency (NUE)	50.92	48.93	0.522
Nitrogen nutrition index (NNI)	1.03	0.77	< 0.001
Nitrogen partial productivity (NPFP)	48.05	27.86	0.037
